# Application of CT texture analysis to assess the localization of primary aldosteronism

**DOI:** 10.1038/s41598-020-57427-7

**Published:** 2020-01-16

**Authors:** Hiroyuki Akai, Koichiro Yasaka, Akira Kunimatsu, Kuni Ohtomo, Osamu Abe, Shigeru Kiryu

**Affiliations:** 1 Department of Radiology, Institute of Medical Science, University of Tokyo, 4-6-1 Shirokanedai, Minato-ku, Tokyo 108-8639 Japan; 2 International University of Health and Welfare, 2600-1 Kitakanemaru, Ohtawara City, Tochigi 324-8501 Japan; 3 Department of Radiology, Graduate School of Medicine, University of Tokyo, 7-3-1 Hongo, Bunkyo-ku, Tokyo 113-8655 Japan; 4 Department of Radiology, International University of Health and Welfare Hospital, 537-3 Iguchi, Nasushiobara, Tochigi 329-2763 Japan

**Keywords:** Predictive markers, Adrenal gland diseases

## Abstract

We performed present study to investigate whether the localization of primary aldosteronism (PA) can be predicted using quantitative texture analysis on unenhanced computed tomography (CT). Plain CT data of 82 PA patients (54 unilateral (right-sided:left-sided = 24:30), 28 bilateral) were analyzed retrospectively. After semi-automatically setting the region of interest to include the whole adrenal gland, texture analyses were performed with or without a Laplacian of Gaussian filter with various spatial scaling factors (SSFs). Logistic regression analysis was performed using the extracted histogram-based texture features to identify parameters capable of predicting excessive aldosterone production. The result of adrenal venous sampling served as gold standard in present study. As a result, logistic regression analysis indicated that the mean gray level intensity (*p* = 0.026), the mean value of the positive pixels (*p* = 0.003) in the unfiltered image, and entropy (*p* = 0.027) in the filtered image (SSF: 2 mm) were significant parameters. Using the model constructed by logistic regression analysis and the optimum cutoff value, the localization of PA (three multiple choices of left, right or bilateral) was determined with an accuracy of 67.1% (55/82). CT texture analysis may provide a potential avenue for less invasive prediction of the localization of PA.

## Introduction

Primary aldosteronism (PA) is a disorder in which aldosteronesynthesis is disproportionately high given the sodium status, relatively autonomous of the major regulators of secretion loading^1^. PA is recognized as the most frequent cause of secondary hypertension^2,3^, and has been reported to account for >5%, and possibly >10%, of hypertension cases in both unselected and referred hypertensive patients^4–6^.

Treatment of PA basically depends on the localization of the disease^1^. For patients with unilateral PA, unilateral laparoscopic adrenalectomy is recommended, while medical treatment (mainly using mineralocorticoid receptor antagonists) is recommended for bilateral PA patients. As it is a straightforward diagnostic test, adrenal venous sampling (AVS) is considered as the gold standard in differentiation of these subtypes of PA^7–9^. However, as it is both technically challenging and invasive, AVS is not routinely used even in some major referral centers^10^. Although less invasive approaches, such as computed tomography (CT), magnetic resonance imaging and biochemical tests, are desirable, these alternative methods lack reliability for distinguishing between unilateral and bilateral disease^7,11,12^. Recently the 11C-metomidate PET-CT scanning is reported to show relatively high diagnostic performance^13^, it is not yet clinically widely available^14^.

Quantitative texture analysis has recently gained much attention for assessing the internal heterogeneity of the targeted organ or tumor^15–17^. Among those analysis, we now can investigate fine to coarse textures of an object by a method involving histogram analyses of images with and without filtration^18^. These textural features are known to provide information of clinically importance not only for tumor characterization^19,20^, but also for the diseased organ itself^21,22^.

We performed present study to investigate whether the localization of PA can be predicted using quantitative texture analysis on unenhanced CT.

## Methods

This study was approved by the Research Ethics Committee of University of Tokyo Hospital as a retrospective medical imaging data analysis using texture analysis and deep learning technique. The requirement for informed consent was waived by the Committee (approval number 11755). All experiments were performed in accordance with the relevant guidelines and regulations.

### Patients

**Figure 1:**
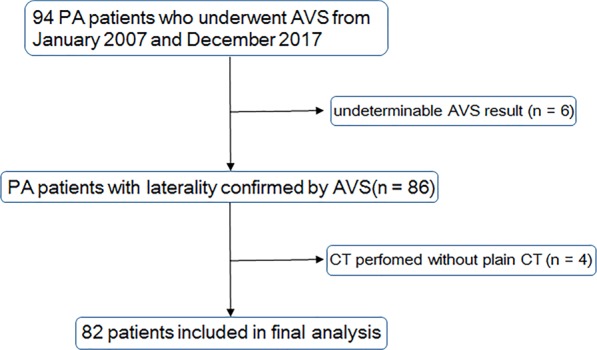
Flowchart of the process of inclusion in study groups. Numbers in parentheses indicate the number of patients.

The study population initially consisted of 94 consecutive patients diagnosed as having PA biochemically, and who underwent AVS to determine the localization of excessive aldosterone production between January 2007 and December 2017. Six patients were excluded due to indeterminate AVS results. A further two patients for whom the interval between CT examination and AVS was more than 1 year were also excluded. Four patients were additionally excluded because a CT protocol without plain CT was performed (Fig. 1). The final study population consisted of 82 patients: 51 men (mean age, 50.3 years; range: 27–69 years) and 31 women (mean age, 50.4 years; range: 34–65 years). The median interval between CT examination and AVS was 58 days (lower – upper quartile: 14–103 days).

### Determination of the localization by AVS

In all cases, AVS was performed with adrenocorticotropic hormone stimulation. During AVS, both plasma aldosterone concentration (PAC) and cortisol concentration were determined in blood collected from the adrenal veins, and in blood collected simultaneously from the inferior vena cava (IVC). The levels of cortisol were used in the calculation of the aldosterone/cortisol ratio ([A/C]). The diagnostic criteria used in this study were as follows: (1) PAC _dominant_ > 1,400 ng/dL; (2) lateralization index ([A/C _dominant_]/A/C _non-dominant_]) > 3 and; (3) contralateral suppression index ([A/C _non-dominant_]/[A/C _IVC_]) < 1^23,24^. If all three criteria were fulfilled, the patient was judged to have unilateral PA; otherwise, a diagnosis of bilateral PA was made.

### CT examination

CT studies were performed using a 64-detector row CT scanner (LightSpeed VCT or Discovery CT750 HD; GE Medical Systems, Milwaukee, WI, USA: number of cases = 6 and 15, respectively) or a 64-, 80-, or 320-detector row CT scanner (Aquillion64, Aquillion Prime, and Aquillion ONE, respectively; Toshiba Medical Systems Corp., Tochigi, Japan: number of cases = 48, 2, and 11, respectively). Although CT was performed with various protocols, the scanning parameters of unenhanced CT were constant, as follows: voltage, 120 kVp; tube current controlled by automatic exposure control technique; matrix, 512 × 512; field of view, 350–450 mm; and slice thickness, 5 mm. Iterative reconstruction methods were not used in the present study.

### Texture analysis

CT texture was analyzed using commercial software (TexRAD; TexRAD Ltd., part of Feedback Plc., Cambridge, UK). The images were evaluated by a single radiologist with 24 years of experience who was blinded to the clinical outcome. The region of interest (ROI) for each adrenal gland was drawn automatically by the software after the observer set a seed on the adrenal gland. If the automatically drawn ROI included adjacent organs or structures, the observer manually amended the ROI to exclude such organs and structures. For each adrenal gland, three consecutive CT slices, including the maximum cross-section of the adrenal gland, were selected for the analysis. Unless the CT slice with the maximum cross-section of the adrenal gland was at the top or bottom of the CT slices including the adrenal gland, the CT slice with the maximum cross-section of the adrenal gland was selected as the center of the three slices.

**Figure 2:**
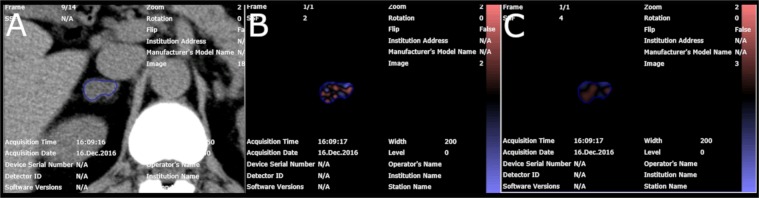
Screen shot of the CT texture analysis software. A polygonal ROI was drawn on the tumor (**A**). Processed images using Laplacian of Gaussian filters with SSF of 2 mm (**B**) and 4 mm (**C**) were automatically generated. The images were displayed using a red or blue scale showing negative or positive pixels, respectively.

Quantitative texture analysis using the software mentioned above was performed using the filtration-histogram technique. First, filtration was performed using a Laplacian of Gaussian band-pass filter to extract features of different sizes with various spatial scaling factors (SSFs). SSFs of 2 and 4 mm were used in this study to reflect medium and coarse textures, respectively (Fig. 2). In the second step, the unfiltered (SSF = 0) and filtered (SSF = 2 and 4 mm) texture features were quantified. The parameters analyzed were the mean gray level intensity (mean), standard deviation (SD), mean value of the positive pixels (mpp), entropy, kurtosis, and skewness^15,18^. Hereafter, these parameters are described by addition of the SSF values after the parameter name. For example, the mean of the positive value pixels of the SSF 2 mm image is referred to as mpp2. A total of 18 parameters were assessed in the present study.

### Statistical analysis

All statistical analyses were performed using EZR software (Saitama Medical Center, Jichi Medical University, Japan)^25^. This software is a graphical user interface for R version 3.2.2 (R Foundation for Statistical Computing, Vienna, Austria). Since the PAC and [A/C] were not normally distributed, spearman rank correlation test was used to assess the correlation of each texture parameters and the result of AVS (PAC and [A/C]). For the estimation of the subtypes of PA, first, we compared the CT texture parameters between the adrenal gland with and without excessive aldosterone production on a per-lesion basis using Student’s *t* test. Then, to select the best combination of parameters to predict the aldosterone production status of each adrenal gland, logistic regression analysis with a stepwise procedure was performed for 5 parameters with the smallest *p* value in the first step. This number of parameters was selected due to the number of adrenal glands without excessive aldosterone production (n = 54). Finally, as the purpose of the present study was to determine the localization of excessive aldosterone production on a per-patient basis, we subtracted the results of logistic regression analysis (i.e., estimated probability) from the right and left adrenal glands, and sought to determine the optimum cutoff value. In all analyses, *p* < 0.05 was taken to indicate statistical significance.

## Results

### Results of AVS

Among the total population of 82 patients, 54 had unilateral PA (right-sided:left-sided = 24:30) and 28 had bilateral PA. For the unilateral PA patients, PAC_dominant_ (median: 4595 ng/dL, lower - upper quartile: 3218–10400 ng/dL) and [A/C_dominant_] (median: 9.92, lower - upper quartile: 5.79–17.5) were very high compared to PAC_non-dominant_ (median: 416 ng/dL, lower - upper quartile: 325–649 ng/dL)and [A/C_non-dominant_] (median: 0.70, lower - upper quartile: 0.54–1.14), respectively. For the bilateral PA patients, median of PAC was 2905 ng/dL (lower - upper quartile: 1753–4275 ng/dL) and median of [A/C] was 2.98 (lower - upper quartile: 2.01–5.14).

### Correlation of texture parameters and AVS result

On CT, the median of the maximum cross-sectional area of the adrenal gland was 117 mm^2^ (lower - upper quartile: 95–163 mm^2^) for the right adrenal gland and 137 mm^2^ (lower -upper quartile: 106–185 mm^2^) for the left adrenal gland. Mean0 (*ρ* = −0.158, p < 0.05), mpp0 (*ρ* = −0.212, p < 0.01) and mean2 (*ρ* = −0.207, p < 0.01) showed statistically significant but weak negative correlation with PAC, And for [A/C], mean2 (*ρ* = −0.261, p < 0.001) showed negative correlation and entropy2 (*ρ* = 0.171, p < 0.05) and kurtosis2 (*ρ* = 0.163, p < 0.05) showed weak positive correlation.

### Estimation of PA subtypes

The results of univariate analysis on a per-lesion basis are shown in Table 1. Mean2 and mpp0 were significantly lower, while entropy2 was significantly higher, in adrenal glands with excessive aldosterone production. The 5 texture parameters with smallest *p* value were mean0, mean2, mpp0, entropy2, and entropy4; logistic regression analysis was performed using these parameters and mpp0 (*p* = 0.003), mean0 (*p* = 0.026), and entropy2 (*p* = 0.027) were found to be significant parameters. The model was determined as follows:

$${\rm{Logit}}=-\,3.85633+0.09773\times {\rm{mean}}0-0.2314\times {\rm{mpp}}0+1.92463\times {\rm{entropy}}2$$

By subtracting the results of the abovementioned model of the left adrenal gland from the right (Logit _individual_ = Logit _right adrenal gland_ − Logit _left adrenal gland_), the optimum cutoff value was found to be 0.25 (i.e., judge as left PA if Logit _individual_ < −0.25, bilateral PA if −0.25 ≤ Logit _individual_ ≤ 0.25, and right PA if 0.25 < Logit _individual_). Using this cutoff value, the accuracy of the testing (three multiple choices of left, right or bilateral) was 67.1% (55/82).

**Table 1:** Texture parameters of adrenal gland in unenhanced CT.

Parameter and SSF	With hypersecretion	Without hypersecretion	Comparison (p value)
**Mean**			
0	14.1 ± 8.67	16.8 ± 10.2	0.082*
2	2.15 ± 2.05	2.92 ± 1.93	0.023**
4	3.89 ± 2.49	4.45 ± 2.55	0.179
**Mpp**			
0	23.2 ± 5.15	25.5 ± 5.09	0.006**
2	32.4 ± 7.51	33.6 ± 8.56	0.392
4	23.6 ± 7.81	22.7 ± 7.74	0.504
**SD**			
0	20.1 ± 3.04	20.8 ± 3.51	0.205
2	40.3 ± 8.91	41.5 ± 10.6	0.441
4	26.0 ± 8.30	24.9 ± 8.39	0.446
**Entropy**			
0	4.10 ± 0.14	4.07 ± 0.16	0.172
2	4.55 ± 0.20	4.48 ± 0.25	0.034**
4	4.18 ± 0.29	4.08 ± 0.36	0.063*
**Kurtosis**			
0	0.39 ± 0.53	0.33 ± 0.55	0.530
2	−0.03 ± 0.56	−0.12 ± 0.42	0.250
4	−0.59 ± 0.41	−0.66 ± 0.37	0.315
**Skewness**			
0	−0.34 ± 0.25	−0.41 ± 0.24	0.089*
2	−0.17 ± 0.29	−0.22 ± 0.27	0.281
4	−0.01 ± 0.33	−0.08 ± 0.33	0.145

All values are shown as mean ± standard deviation. * are the parameters with p < 0.1, and **p < 0.0.

## Discussion

In the present study, we investigated whether the localization of PA can be predicted using quantitative texture analysis on unenhanced CT. The results indicated that mpp0, mean0, and entropy2 were significant parameters, and the model constructed by logistic regression analysis determined the localization of PA with an accuracy of 67.1%.

The accuracy of adrenal CT for localization of the source of excessive aldosterone secretion was reported to be relatively low. In one study of 41 PA patients with AVS, the concordance rate between CT and AVS was only 54%^12^. In another study including a larger number of PA patients (203 patients with both CT and AVS), CT correctly identified unilateral or bilateral disease in only 53% of cases^7^. In the same paper, it was reported that 22% patients would have been incorrectly excluded as candidates for adrenalectomy, and 25% may have had unnecessary or inappropriate surgery based on CT findings. Although the accuracy achieved (67.1%) was not satisfactory, CT texture analysis extracted some informative features from unenhanced CT, resulting in greater accuracy compared to previous reports.

Of the 18 texture features obtained, mpp of the unfiltered image showed the strongest correlation with the status of aldosterone production by the adrenal gland, and adrenal glands with hypersecretion of aldosterone tended to show lower mpp0 values. We speculated that the lower mpp0 value indicated the presence of aldosterone-producing adenoma regardless of whether a tumor was apparent or not. In addition, the difference between the mean and mpp of the unfiltered image was due only to the inclusion or exclusion of negative value pixels. The observation that mpp0 showed the strongest correlation suggests that elimination of negative value pixels may yield better accuracy when analyzing small structures and those close to the bone. Such structures usually suffer from a partial volume effect and streak artifacts: exclusion of the negative value pixels may filter out the outliers from such artifacts.

In the direct comparison of texture features and AVS result, not only the features that were used in final model for estimating PA subtypes but also other features such as mean2 and kurtosis2 showed statistically significant correlation with PAC and [A/C]. On contrast, no features using medium filter (SSF: 4 mm) showed significant correlation with measured value of AVS. We speculate that the medium filter was too large to adequately assess the internal heterogeneity of the adrenal glands, and the fine filter (SSF: 2 mm) is proper in assessing the small structure like adrenal gland. There can be a possibility that smaller size of filter (1–2 mm) is more beneficial in assessing adrenal glands, we could not check the possibility since the used software in the present study does not allow smaller filter than 2 mm.

There were several limitations to the present study. First, due to the long study period, different CT scanners were used. However, Yasaka *et al*. reported that most CT texture features were robust among different scanners, except for skewness and kurtosis in filtered images featuring medium and coarse textures^26^. Second, due to the variety of CT scanning protocols, we could not include data of enhanced images. Further investigations are required to determine whether these images are useful. In addition, the study population was relatively small, so we could not perform validation testing. Future studies in larger numbers of patients are necessary to validate our results.

In conclusion, the present study showed that there were correlations between some of the texture features of plain CT and the aldosterone production state of the adrenal gland in PA patients. CT texture analysis may provide a less invasive means of predicting the localization of PA. By searching more adequate size of filtering and combination with more complicated features (e.g. second order features), CT texture analysis may further accurately assess the internal heterogeneity of the adrenal glands, resulting in better prediction of the localization of PA.

## Data Availability

The datasets generated during and/or analyzed during the current study are available from the corresponding author on reasonable request.
